# Modification effect of ideal cardiovascular health metrics on genetic association with incident heart failure in the China Kadoorie Biobank and the UK Biobank

**DOI:** 10.1186/s12916-021-02122-1

**Published:** 2021-10-22

**Authors:** Ruotong Yang, Jun Lv, Canqing Yu, Yu Guo, Pei Pei, Ninghao Huang, Ling Yang, Iona Y. Millwood, Robin G. Walters, Yiping Chen, Huaidong Du, Ran Tao, Junshi Chen, Zhengming Chen, Robert Clarke, Tao Huang, Liming Li, on behalf of the China Kadoorie Biobank Collaborative Group, Junshi Chen, Zhengming Chen, Robert Clarke, Rory Collins, Yu Guo, Liming Li, Jun Lv, Richard Peto, Robin Walters, Daniel Avery, Ruth Boxall, Derrick Bennett, Yumei Chang, Yiping Chen, Zhengming Chen, Robert Clarke, Huaidong Du, Simon Gilbert, Alex Hacker, Mike Hill, Michael Holmes, Andri Iona, Christiana Kartsonaki, Rene Kerosi, Ling Kong, Om Kurmi, Garry Lancaster, Sarah Lewington, Kuang Lin, John McDonnell, Iona Millwood, Qunhua Nie, Jayakrishnan Radhakrishnan, Paul Ryder, Sam Sansome, Dan Schmidt, Paul Sherliker, Rajani Sohoni, Becky Stevens, Iain Turnbull, Robin Walters, Jenny Wang, Lin Wang, Neil Wright, Ling Yang, Xiaoming Yang, Yu Guo, Xiao Han, Can Hou, Jun Lv, Pei Pei, Chao Liu, Canqing Yu, Zengchang Pang, Ruqin Gao, Shanpeng Li, Shaojie Wang, Yongmei Liu, Ranran Du, Yajing Zang, Liang Cheng, Xiaocao Tian, Hua Zhang, Yaoming Zhai, Feng Ning, Xiaohui Sun, Feifei Li, Silu Lv, Junzheng Wang, Wei Hou, Mingyuan Zeng, Ge Jiang, Xue Zhou, Liqiu Yang, Hui He, Bo Yu, Yanjie Li, Qinai Xu, Quan Kang, Ziyan Guo, Dan Wang, Ximin Hu, Jinyan Chen, Yan Fu, Zhenwang Fu, Xiaohuan Wang, Min Weng, Zhendong Guo, Shukuan Wu, Yilei Li, Huimei Li, Zhifang Fu, Ming Wu, Yonglin Zhou, Jinyi Zhou, Ran Tao, Jie Yang, Jian Su, Fang Liu, Jun Zhang, Yihe Hu, Yan Lu, Liangcai Ma, Aiyu Tang, Shuo Zhang, Jianrong Jin, Jingchao Liu, Zhenzhu Tang, Naying Chen, Ying Huang, Mingqiang Li, Jinhuai Meng, Rong Pan, Qilian Jiang, Jian Lan, Yun Liu, Liuping Wei, Liyuan Zhou, Ningyu Chen Ping Wang, Fanwen Meng, Yulu Qin, Sisi Wang, Xianping Wu, Ningmei Zhang, Xiaofang Chen, Weiwei Zhou, Guojin Luo, Jianguo Li, Xiaofang Chen, Xunfu Zhong, Jiaqiu Liu, Qiang Sun, Pengfei Ge, Xiaolan Ren, Caixia Dong, Hui Zhang, Enke Mao, Xiaoping Wang, Tao Wang, Xi Zhang, Ding Zhang, Gang Zhou, Shixian Feng, Liang Chang, Lei Fan, Yulian Gao, Tianyou He, Huarong Sun, Pan He, Chen Hu, Xukui Zhang, Huifang Wu, Pan He, Min Yu, Ruying Hu, Hao Wang, Yijian Qian, Chunmei Wang, Kaixu Xie, Lingli Chen, Yidan Zhang, Dongxia Pan, Qijun Gu, Yuelong Huang, Biyun Chen, Li Yin, Huilin Liu, Zhongxi Fu, Qiaohua Xu, Xin Xu, Hao Zhang, Huajun Long, Xianzhi Li, Libo Zhang, Zhe Qiu

**Affiliations:** 1 Department of Epidemiology & Biostatistics, School of Public Health, Peking University Health Science Center, Peking University, 38 Xueyuan Road, Beijing, 100191 China; 2 Peking University Center for Public Health and Epidemic Preparedness & Response, Beijing, 100191 China; 3 Key Laboratory of Molecular Cardiovascular Sciences (Peking University), Ministry of Education, Beijing, China; 4 Chinese Academy of Medical Sciences, Beijing, China; 5 Medical Research Council Population Health Research Unit at the University of Oxford, Oxford, United Kingdom; 6 Clinical Trial Service Unit & Epidemiological Studies Unit (CTSU), Nuffield Department of Population Health, University of Oxford, Oxford, United Kingdom; 7 Institute of Chronic Disease, Jiangsu Provincial Center for Disease Control and Prevention, Jiangsu, China; 8 China National Center for Food Safety Risk Assessment, Beijing, China; 9 Center for Intelligent Public Health, Academy for Artificial Intelligence, Peking University, Beijing, 100191 China

**Keywords:** Heart failure, Ideal cardiovascular health metrics, Genetic risk

## Abstract

**Background:**

Both genetic and cardiovascular factors contribute to the risk of developing heart failure (HF), but whether idea cardiovascular health metrics (ICVHMs) offset the genetic association with incident HF remains unclear.

**Objectives:**

To investigate the genetic association with incident HF as well as the modification effect of ICVHMs on such genetic association in Chinese and British populations.

**Methods:**

An ICVHMs based on smoking, drinking, physical activity, diets, body mass index, waist circumference, blood pressure, blood glucose, and blood lipids, and a polygenic risk score (PRS) for HF were constructed in the China Kadoorie Biobank (CKB) of 96,014 participants and UK Biobank (UKB) of 335,782 participants which were free from HF and severe chronic diseases at baseline.

**Results:**

During the median follow-up of 11.38 and 8.73 years, 1451 and 3169 incident HF events were documented in CKB and UKB, respectively. HF risk increased monotonically with the increase of PRS per standard deviation (CKB: hazard ratio [*HR*], 1.19; 95% confidence interval [*CI*], 1.07, 1.32; UKB: 1.07; 1.03, 1.11; *P* for trend < 0.001). Each point increase in ICVHMs was associated with 15% and 20% lower risk of incident HF in CKB (0.85; 0.81, 0.90) and UKB (0.80; 0.77, 0.82), respectively. Compared with unfavorable ICVHMs, favorable ICVHMs was associated with a lower HF risk, with 0.71 (0.44, 1.15), 0.41 (0.22, 0.77), and 0.48 (0.30, 0.77) in the low, intermediate, and high genetic risk in CKB and 0.34 (0.26, 0.44), 0.32 (0.25, 0.41), and 0.37 (0.28, 0.47) in UKB (*P* for multiplicative interaction > 0.05). Participants with low genetic risk and favorable ICVHMs, as compared with high genetic risk and unfavorable ICVHMs, had 56~72% lower risk of HF (CKB 0.44; 0.28, 0.70; UKB 0.28; 0.22, 0.37). No additive interaction between PRS and ICVHMs was observed (relative excess risk due to interaction was 0.05 [−0.22, 0.33] in CKB and 0.04 [−0.14, 0.22] in UKB).

**Conclusions:**

In CKB and UKB, genetic risk and ICVHMs were independently associated with the risk of incident HF, which suggested that adherence to favorable cardiovascular health status was associated with a lower HF risk among participants with all gradients of genetic risk.

**Supplementary Information:**

The online version contains supplementary material available at 10.1186/s12916-021-02122-1.

## Background

Heart failure (HF) has become a major clinical and public health challenge worldwide [1–4]. Both genetic and environmental factors play roles in the development of HF. Previous observational studies have provided considerable evidence that a healthy lifestyle pattern has been associated with a lower risk of HF [5–11]. Ideal cardiovascular health metrics (ICVHMs) which additionally include three non-behavioral factors recommended by the American Heart Association (AHA) [12], namely blood pressure, plasma glucose level, and total cholesterol level, have also been shown to provide a protective effect for HF [13].

Furthermore, familial risk of HF suggested a genetic predisposition [14–17] and genome-wide association studies (GWAS) have been successful in identifying several genetic variants associated with HF [18–21]. The genetic risk of cardiovascular disease has been found to be partially offset by healthy lifestyle factors [22–25]; however, little is known about the role and strength that ICVHMs plays in the association of genetic factors with incident HF.

Therefore, in the present study, we constructed a polygenic risk score (PRS) based on previous GWAS to investigate the strength of genetic association with incident HF and further established an ICVHMs to investigate possible interactions between ICVHMs and PRS in 2 independent prospective cohorts of the China Kadoorie Biobank (CKB) and the UK Biobank (UKB).

## Methods

### Study population

The CKB and UKB study design, protocol, procedures, and characteristics have been described in detail previously [26–28]. In brief, 512,725 participants aged 35 to 79 years in 5 urban and 5 rural areas across China during 2004–2008 and 502,505 participants aged 40 to 70 years at 22 assessment centers throughout the UK between 2006 and 2010 were included in the baseline survey of CKB and UKB, respectively. Participants completed extensive baseline questionnaires, interviews, and physical measurements, and their blood samples were collected for genotyping. All participants provided written informed consent for the 2 studies. Ethics approvals for the CKB study were obtained from the Ethical Committee of the Chinese Center for Disease Control and Prevention (Beijing, China), the Oxford Tropical Research Ethics Committee, and the University of Oxford (UK). The UKB study has approval from the National Information Governance Board for Health and Social Care and the National Health Service North West Multicenter Research Ethics Committee.

### Genotyping and imputation

In CKB, single nucleotide polymorphisms (SNPs) were genotyped in 95,680 individuals using a 384-SNP Illumina GoldenGate® array and in 32,410 participants using a custom Affymetrix Axiom® 700K variant array at BGI laboratory. Individuals with low call rate, sex mismatch, heterozygosity *F* statistic SD score ≥5, Hardy–Weinberg disequilibrium, or duplication of genetic data were excluded. A total of 100,640 participants with genetic data were included in this study.

In UKB, participants were genotyped using the UKB Lung Exome Variant Evaluation Axiom (*n* = 49,949) and the UKB Axiom array (*n* = 452,713). Imputed genotype data were based on merged UK10K and 1000 Genomes phase 3 panels. Participants with excess heterozygosity or genetic and reported gender mismatches were excluded. Finally, we included 487,298 participants with genetic data.

### Polygenic risk score

We derived a PRS from 15 SNPs that achieved genome-wide significance for association with HF in discovery GWAS in the European population (Additional file 1: Table S1) [20, 21]. In CKB, due to natural genetic background differences between Asian and European populations and no GWAS studies of heart failure in Asian populations, PRS was constructed based on 2 SNPs (rs17042102 and rs4746140) that were significantly associated with HF (*P* < 0.05) in CKB (Additional file 1: Table S2). In UKB, all 15 SNPs for HF were used to construct the PRS. In both 2 cohorts, the number of associated alleles at each SNP for each individual was summed after multiplication with the effect size between the SNP and HF to generate a PRS. We used logistic regression analysis to calculate Effron pseudo-*R*^2^ value to analyze the explanatory variance of PRSs on the prevalence of heart failure in the two studies (adjusted for age and sex). And the results were pseudo *R*^2^ =10.8%, *P* < 0.001 in CKB and pseudo *R*^2^ = 7.1%, *P* <0.001 in UKB. The scores were then categorized into low (lowest tertile), intermediate (second tertile), and high (highest tertile) risk.

### Ideal cardiovascular health metrics

Information on lifestyle factors (smoking, alcohol consumption, physical activity, diet, and obesity) and other health-related measurements (blood pressure, blood glucose, and blood lipid) were obtained through baseline questionnaires, interviews, and physical measurements. Each factor used to construct ICVHMs was dichotomized into the healthy group and unhealthy group, and the details are shown in Additional file 1: Table S3.

Specifically, in CKB, the healthy smoking group was defined as no current smoking and excluded those who had stopped smoking due to illness. The healthy alcohol drinking group included never-regular, weekly, and moderate daily drinkers (i.e., drinking ≤ 25 g of pure alcohol in men and ≤15 g in women per day) which was consistent with the Chinese dietary guidelines [29]. Eating vegetables and fruits daily and red meat on 1 to 6 days a week was considered a healthy diet [30, 31]. For physical activity, those who engaged in a sex-specific median or higher level of physical activity constituted the healthy group. For obesity indexes, the healthy group was defined as normal body mass index (*BMI*; 18.5 ≤ *BMI* <24.0 kg/m^2^) and normal waist circumference (*WC*; *WC* < 90 cm for men and *WC* < 85 cm for women) according to the Chinese standard. The ideal blood pressure was defined as untreated blood pressure <120/80 mmHg without a self-reported diagnosis of hypertension [32]. The ideal blood glucose group excluded those with a self-reported history of diabetes and was further defined as a random blood glucose level <5.6 mmol/l and fasting time ≥8 h, a blood glucose level <11.1 mmol/l and fasting time <8 h, or a fasting blood glucose level <5.6 mmol/l. Lacking lipid information in the baseline survey of CKB, the ideal lipid group was defined as those currently not taking lipid-lowering medication.

In UKB, the healthy smoking group was defined as no current smoking. Moderate alcohol consumption (women >0 and ≤14g/day; men >0 and ≤28g/day) was considered healthy. Healthy physical activity was defined as ≥150 min moderate activity per week or ≥75 min vigorous activity per week or equivalent combination or moderate physical activity at least 5 days a week or vigorous activity once a week. Consistent with the current recommendation [30] and previous studies [25, 33], a healthy diet was defined as vegetables and fruits >3 servings per day, processed meat ≤1 serving per day, and unprocessed meat ≤1.5 serving per day. For obesity indexes, the healthy group was defined as normal *BMI* (18.5 ≤ *BMI* <25.0 kg/m^2^) and normal *WC* (*WC* < 102 cm for men and *WC* < 88 cm for women). Three ideal metabolic factors were defined as untreated blood pressure <120/80 mmHg, fasting blood glucose <100 mg/dl, and untreated total cholesterol <200 mg/dl, respectively.

The ICVHMs range from 0 (unhealthiest) to 8 (healthiest) and were categorized as favorable (6 to 8), intermediate (3 to 5), and unfavorable (0 to 2).

### Covariates

Covariates for the primary analyses in CKB involved sociodemographic characteristics (age, sex, education [illiterate and elementary, middle school, high school and above], socioeconomic status [annual income <10 000, 10 000–19 999, and ≥20 000 yuan], and family medical history) recorded at baseline.

In UKB, education was categorized as higher (college/university degree or other professional qualification), upper secondary (second/final stage of secondary education), lower secondary (first stage of secondary education), vocational (work-related practical qualifications), or others. Socioeconomic status categories were derived from Townsend deprivation index quintiles 1, 2 to 4, and 5. The first 20 principal components of ancestry were also considered as covariates for the primary analyses.

### Heart failure diagnosis

In CKB, incident cases of HF occurring during follow-up were identified by linkage to death and disease registries and national health insurance databases, using participant’s unique national identification number. HF cases were coded using the I50 code from the Tenth Revision of the International Classification of Diseases (ICD-10).

In UKB, incident HF was identified from hospital inpatient records and the diagnoses were obtained from the Hospital Episode Statistics for England, Scottish Morbidity Record data for Scotland, and the Patient Episode Database for Wales. Death was ascertained via linkage to death registries. Diagnoses were recorded using the ICD coding system.

### Statistical analyses

Participants in CKB were followed up until the date of the first diagnosis of HF, death, loss to follow-up, or December 31, 2017, whichever came first. Participants in UKB were followed up until the date of the first diagnosis, death, loss to follow-up, or the last date of hospital admission (March 31, 2017, for England; October 31, 2016, for Scotland; and February 29, 2016, for Wales), whichever came first.

Baseline characteristics of the participants were described by the incidence of HF summarized as percentage for categorical variables and means with standard deviation (*SD*) for continuous variables. We constructed Kaplan–Meier incidence curves of PRS categories according to different ICVHMs categories in relation to HF and the log-rank tests were calculated. Cox proportional hazard regression models were used to estimate the hazard ratio (*HR*) and 95% confidence interval (*CI*) for the association of PRS categories, ICVHMs categories, and the combination of PRS and ICVHMs categories (9 categories with high genetic risk and unfavorable ICVHMs as reference) with incident HF. For CKB, the models were adjusted for sex, education, marital status, menopausal status (only in women), and family history of heart attack or stroke and stratified by region and age at baseline (in 5-year intervals). For UKB, the models were adjusted for sex, education, socioeconomic status, and the first 20 principal components and stratified by age at baseline (in 5-year intervals). The proportional hazard assumption was examined using the Schoenfeld residual technique and satisfied. Incidence rates per 1000 person-years were calculated.

In the number of HF events between given groups over a 10-year period, we calculated the cumulative risk as the incidence of HF for ICVHMs categories and the absolute risk reduction with the unfavorable ICVHMs as the reference group in different PRS categories. The *95% CI*s for the absolute risk reduction were derived by drawing 1000 bootstrap samples from the estimation dataset.

In addition, we used Cox regression to test the interactions between ICVHMs and PRS adjusted as previously mentioned in CKB and UKB. The likelihood ratio test was used to compare the models with and without cross product-terms. We also calculated relative excess risk due to interaction using the following formula: *RERI* = *RR*_11_ − *RR*_10_ − *RR*_01_ + 1, and attributable proportion due to interaction (*AP* = *RERI*/*RR*_11_) to test the additive interaction [34]. *P* values were 2-sided with a threshold for significance set at less than 0.05. All analyses were performed using Stata version 16 (StataCorp).

## Results

### Population characteristics

From the 100,640 individuals with available genotypes in CKB, participants with a prior medical history of heart disease (*n* = 3084), stroke (*n* = 1400), or cancer (*n* = 421) and missing data for BMI (*n* = 1) were excluded, leaving a total of 96,014 participants. From the 487,298 individuals in UKB, participants with missing data on lifestyle factors (*n* = 18,962), biochemical indicators (*n* = 87,886), TDI or education (*n* = 6848), and participants with HF (*n* = 2168), coronary heart disease (*n* = 19,917), stroke (*n* = 2582), or cancer (*n* = 35,035) at baseline were excluded, leaving 335,782 participants for the current analysis (Table 1). During the median follow-up of 11.38 and 8.73 years, 1451 and 3169 incident HF events were documented in CKB and UKB participants with genetic data, respectively. In both cohorts, most participants engaged in the intermediate ICVHMs (3 to 5).

**Table 1:** Characteristics of participants with genetic information in CKB and UKB

No. (%)^a^	CKB		UKB	
Characteristic	No incident HF (*n* = 94,563)	Incident HF (*n* = 1 451)	No incident HF (*n* = 332,613)	Incident HF (*n* = 3169)
Age, mean (SD), y	52.7 (10.9)	62.7 (8.9)	56.0 (8.1)	61.8 (6.2)
Sex				
Male	40,395 (42.7)	691 (47.6)	152,147 (45.7)	2030 (64.1)
Female	54,168 (57.3)	760 (52.4)	180,466 (54.3)	1136 (35.9)
Socioeconomic status^b^				
Low	29,435 (31.1)	656 (45.2)	66,262 (19.9)	879 (27.8)
Intermediate	28,261 (29.9)	369 (25.4)	199,545 (60.0)	1739 (54.9)
High	36,867 (39.0)	426 (29.4)	66,806 (20.1)	548 (17.3)
Healthy lifestyle factors				
Currently not smoking	65,152 (68.9)	864 (59.6)	298,678 (89.8)	2635 (83.2)
Not excessive drinking	87,302 (92.3)	1346 (92.8)	165,902 (49.9)	1965 (62.1)
Active physical activity	44,662 (47.2)	490 (33.8)	239,307 (72.0)	351 (11.1)
Healthy diet	6264 (6.6)	75 (5.2)	289,401 (87.0)	1365 (43.1)
Normal BMI and WC	47,306 (50.0)	708 (48.8)	109,555 (32.9)	608 (19.2)
Optimal cardiometabolic factors				
Ideal blood pressure	25,319 (26.8)	192 (13.2)	40,554 (12.2)	179 (5.7)
Ideal blood glucose	82,566 (87.3)	1191 (82.1)	285,859 (85.9)	2327 (73.5)
Ideal blood lipid	94,429 (99.9)	1445 (99.6)	106,983 (32.2)	1391 (43.9)
ICVHMs				
Unfavorable	2267 (2.4)	62 (4.3)	14,171 (4.3)	735 (23.2)
Intermediate	66,034 (69.8)	1184 (81.6)	239,771 (72.1)	939 (29.7)
Favorable	26,262 (27.8)	205 (14.1)	78,671 (23.7)	881 (27.8)
Weighted genetic risk category^c^				
Low	40,176 (42.5)	590 (40.7)	118,849 (35.7)	1056 (33.3)
Intermediate	23,117 (24.5)	340 (23.4)	116,045 (34.9)	1122 (35.4)
High	31,270 (33.1)	521 (35.9)	97,719 (29.4)	988 (31.2)

*SD* standard deviation, *BMI* body mass index, *WC* waist circumstance, *ICVHMs* ideal cardiovascular health metrics

^a^Percentages may not sum to 100 because of rounding

^b^Socioeconomic status in CKB means annual income <10,000, 10,000–19,999, and ≥ 20 000 yuan. Socioeconomic status in UKB assessed with the Townsend deprivation index, which combines information on social class, employment, car availability, and housing

^c^Genetic risk categories were defined according to a weighted polygenic risk score as low (lowest tertile), intermediate (intermediate tertile), and high (highest tertile)

### Associations of PRS and ICVHMs with incident heart failure

As shown in Table 2, HF risk increased monotonically with the increase of PRS per standard deviation (CKB: *HR*, 1.19; *95% CI*, 1.07, 1.32; UKB: *HR*, 1.07; *95% CI*, 1.03, 1.11; *P* for trend < 0.001). Participants with high genetic risk (highest tertile of PRS) had a higher risk of incident HF than those with low genetic risk (lowest tertile of PRS) (CKB: 1.15; 1.02, 1.29; UKB: 1.16; 1.06, 1.27). The PRS constructed based on 15 SNPs did not have a significant prospective association (*P* for trend < 0.001) with heart failure in CKB (Additional file 1: Table S4).

**Table 2:** Associations of polygenic risk score and ideal cardiovascular health metrics with risk of incident heart failure in CKB and UKB

	Cases/1000 PYs	*HR* (*95% CI*)^a^	*P* trend^b^
CKB			
Genetic risk (per risk allele)^c^		1.19 (1.07, 1.32)	0.001
Low (*n* = 40,766)	1.35	1.00	
Intermediate (*n* = 23,457)	1.35	1.03 (0.90, 1.17)	
High (*n* = 31,791)	1.53	1.15 (1.02, 1.29)	
ICVHMs (per point)^d^		0.85 (0.81, 0.90)	<0.001
Unfavorable (*n* = 2329)	2.68	1.00	
Intermediate (*n* = 67,218)	1.67	0.76 (0.59, 0.99)	
Favorable (*n* = 26,467)	0.69	0.55 (0.41, 0.74)	
UKB			
Genetic risk (per risk allele)		1.07 (1.03, 1.11)	<0.001
Low (*n* = 119,907)	1.02	1.00	
Intermediate (*n* = 117,168)	1.11	1.08 (0.99, 1.18)	
High (*n* = 98,707)	1.16	1.16 (1.06, 1.27)	
ICVHMs (per point)		0.80 (0.77, 0.82)	<0.001
Unfavorable (*n* = 14,494)	2.60	1.00	
Intermediate (*n* = 242,177)	1.15	0.48 (0.43, 0.54)	
Favorable (*n* = 79,111)	0.64	0.34 (0.29, 0.39)	

^a^Cox proportional hazard regression in CKB adjusted for sex, education, marital status, and family histories of heart attack or stroke at baseline and stratified jointly by study area and age at baseline in the 5-year interval. Cox proportional hazard regression in UKB adjusted for sex, education, marital status, and socioeconomic status and first 20 principal components of ancestry and stratified jointly by age at baseline in the 5-year interval. *HR*, hazard ratio; *CI*, confidence interval

^b^*P* value for trend calculated treating the three scores as continuous variables

^c^Genetic risk categories were defined according to a weighted polygenic risk score as low (lowest tertile), intermediate (intermediate tertile), and high (highest tertile)

^d^*ICVHMs*, ideal cardiovascular health metrics. The categories defined according to ideal cardiovascular health metrics as favorable (6 to 8), intermediate (3 to 5), and unfavorable (0 to 2)

Each point increase in ICVHMs was associated with 15% and 20% lower risk of incident HF in CKB (0.85; 0.81, 0.90; *P* for trend < 0.001) and UKB (0.80; 0.77, 0.82; *P* for trend < 0.001), respectively. Compared with participants with unfavorable ICVHMs, the adjusted *HR*s (*95% CI*s) of those with intermediate and favorable ICVHMs were 0.76 (0.59, 0.99) and 0.55 (0.41, 0.74) in CKB and 0.48 (0.43, 0.54) and 0.34 (0.29, 0.39) in UKB.

### Interplay between ICVHMs and genetic risk on the development of heart failure

**Fig. 1:**
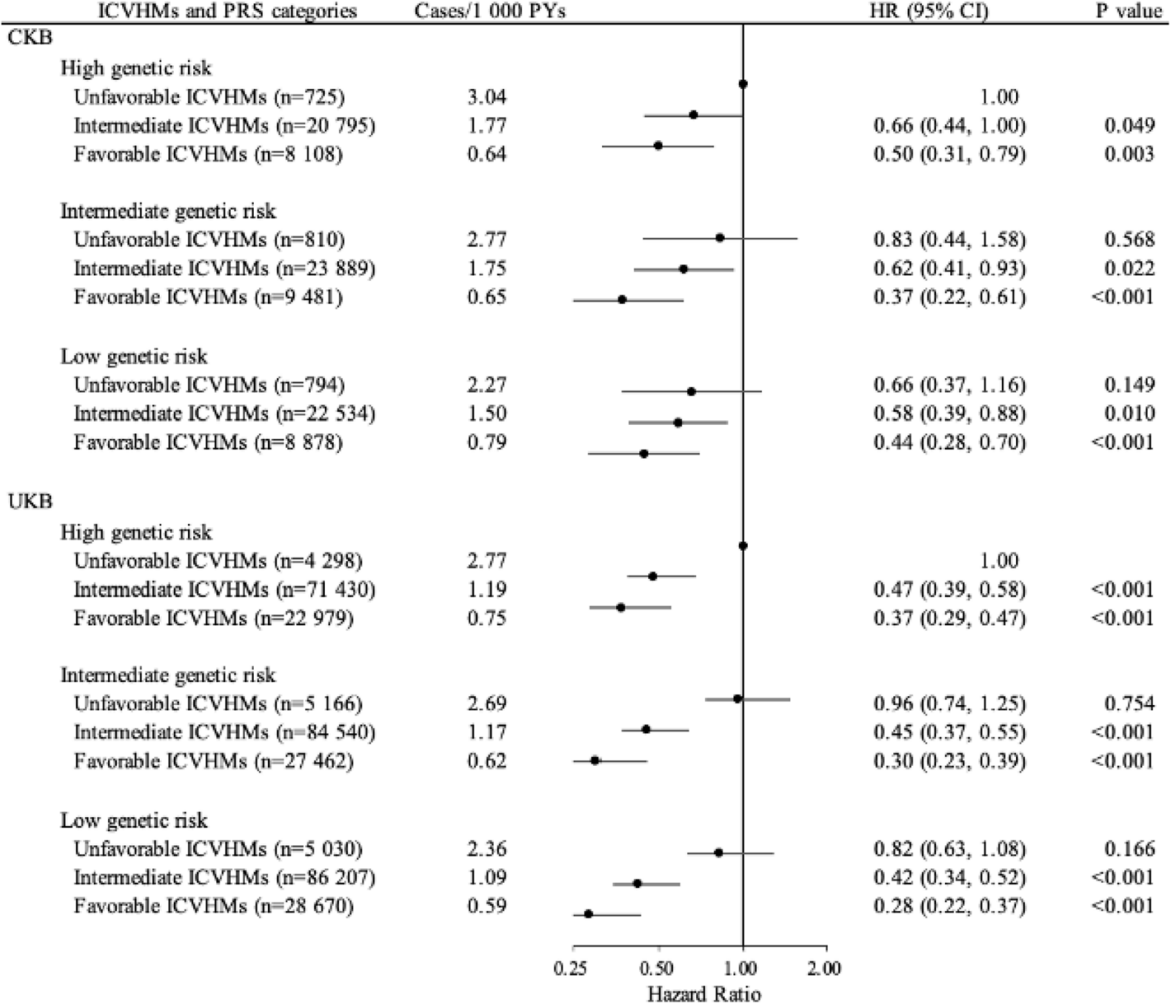
Joint analysis for associations of ideal cardiovascular health metrics categories and genetic risk groups with risk of incident heart failure in CKB and UKB. ICVHMs, ideal cardiovascular health metrics. The categories were defined according to ICVHMs as favorable (6 to 8), intermediate (3 to 5), and unfavorable (0 to 2). PRS, polygenic risk score. The genetic risk categories were defined according to a weighted PRS as low (lowest tertile), intermediate (intermediate tertile), and high (highest tertile). The numbers above the curves represent the hazard ratios and the 95% confidence intervals. Cox proportional hazard regression in CKB adjusted for sex, education, marital status, and family histories of heart attack or stroke at baseline and stratified jointly by study area and age at baseline in the 5-year interval. Cox proportional hazard regression in UKB adjusted for sex, education, marital status, and socioeconomic status and first 20 principal components of ancestry and stratified jointly by age at baseline in the 5-year interval. *HR*, hazard ratio; *CI*, confidence interval

If combining the genetic risk and ICVHMs categories as shown in Fig. 1, joint effects were observed on the risk of incident HF in dose–response manners; the overall risk of incident HF decreased as genetic risks decreased and the number of favorable cardiovascular factors increased. Participants with low genetic risk and favorable ICVHMs, as compared with high genetic risk and unfavorable ICVHMs, had 56% and 72% lower risk of HF in Chinese (*HR*, 0.44; *95% CI*, 0.28, 0.70) and British (*HR*, 0.28; *95% CI*, 0.22, 0.37), respectively (Fig. 1). However, no significant multiplicative interaction between genetic risk and ICVHMs was observed (*P* = 0.616 in CKB and 0.726 in UKB; Additional file 1: Table S5)

**Fig. 2:**
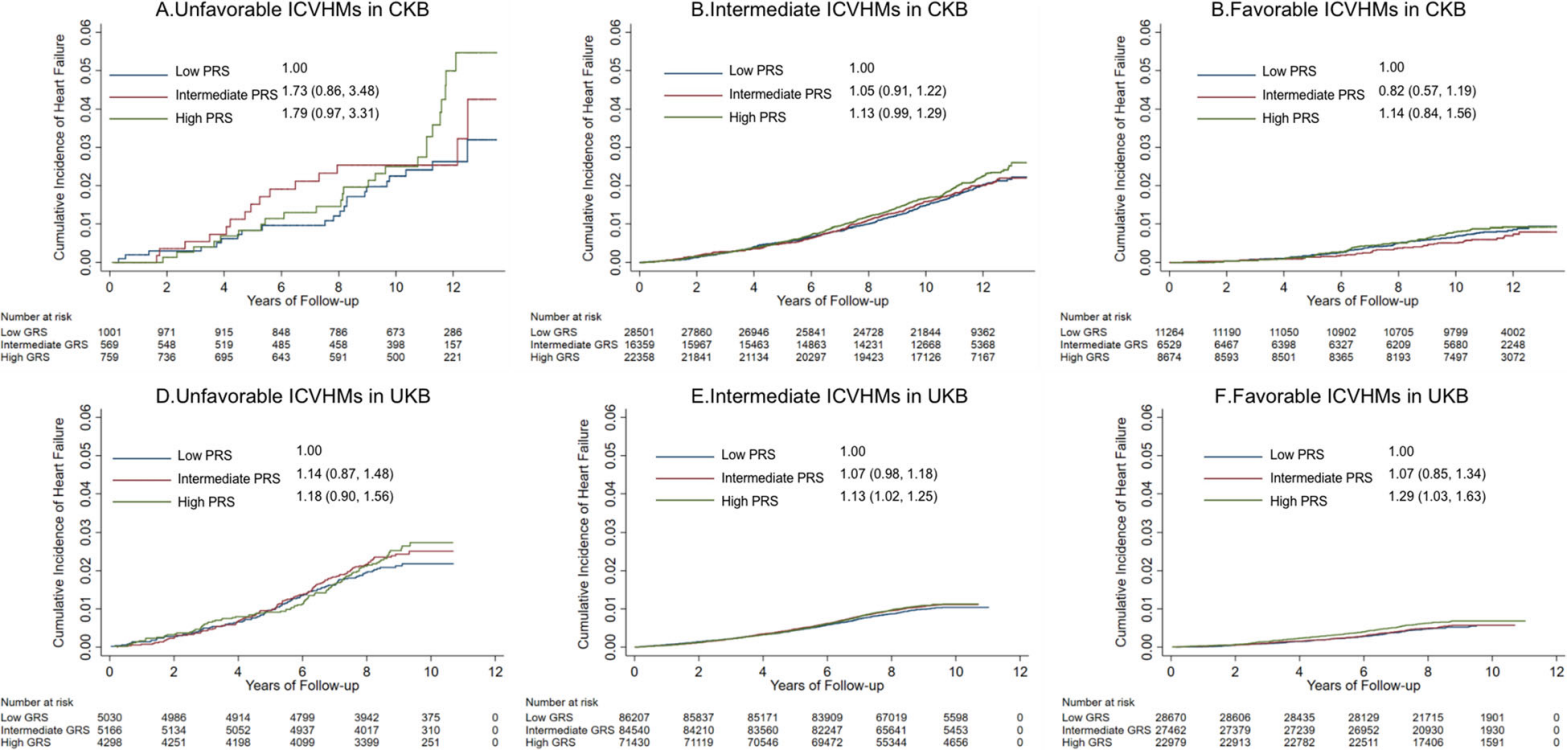
Risk of incident heart failure according to genetic risk categories among different ideal cardiovascular health metrics categories in CKB and UKB. ICVHMs, ideal cardiovascular health metrics. The categories were defined according to ICVHMs as favorable (6 to 8), intermediate (3 to 5), and unfavorable (0 to 2). PRS, polygenic risk score. The genetic risk categories were defined according to a weighted PRS as low (lowest tertile), intermediate (intermediate tertile), and high (highest tertile). The numbers above the curves represent the hazard ratios and the 95% confidence intervals. Cox proportional hazard regression in CKB adjusted for sex, education, marital status, and family histories of heart attack or stroke at baseline and stratified jointly by study area and age at baseline in the 5-year interval. Cox proportional hazard regression in UKB adjusted for sex, education, marital status, and socioeconomic status and first 20 principal components of ancestry and stratified jointly by age at baseline in the 5-year interval. HR, hazard ratio; CI, confidence interval

After stratifying participants by ICVHMs categories (unfavorable, intermediate, and favorable), participants with varying degrees of genetic risk had greater differences in the unfavorable ICVHM groups according to the cumulative incidence curves of HF, although the results of log-rank tests were not significant (*P* > 0.05; Fig. 2). *HR*s and *95% CI*s for high genetic risk compared to low genetic risk in unfavorable ICVHMs were 1.79 (0.97, 3.31) in CKB and 1.18 (0.90, 1.56) in UKB. Analyses stratified by genetic risk categories with unfavorable ICVHMs as the reference groups found that favorable ICVHMs was associated with a lower HF risk in all three genetic risk groups (*HR*s and *95% CI*s in the low, intermediate, and high genetic risk were 0.71 [0.44, 1.15], 0.41 [0.22, 0.77], and 0.48 [0.30, 0.77] in CKB and 0.34 [0.26, 0.44], 0.32 [0.25, 0.41], and 0.37 [0.28, 0.47] in UKB; Table 3). In the low, intermediate, and high genetic risk groups, favorable ICVHMs, as compared with the unfavorable ICVHMs, reduced the risk of HF by 33%, 55%, and 50% in CKB and 66%, 69%, and 63% in UKB, respectively (Fig. 1). Furthermore, among participants with high genetic risk, participants with a favorable ICVHMs have lower HF risk (CKB: *HR*, 0.48; *95% CI*, 0.30, 0.77; UKB: *HR*, 0.37; *95% CI*, 0.28, 0.47) and reduced 1.70% 10-year absolute risk in CKB and 2.06% in UKB, compared with the unfavorable group (Table 3).

**Table 3:** Risk of incident heart failure according to ideal cardiovascular health metrics categories within each genetic risk category in CKB and UKB

ICVHMs and PRS categories	Cases/1000 PYs	*HR* (*95% CI*)^a^	*P* value^b^	Absolute risk over 10 years (*95% CI*)	Absolute risk reduction over 10 years
CKB					
High genetic risk^c^					
ICVHMs^d^ per point		0.86 (0.79, 0.94)	0.003		
Unfavorable ICVHMs (*n* = 725)	3.04	1.00		2.50% (1.53, 4.06)	
Intermediate ICVHMs (*n* = 20,795)	1.77	0.64 (0.42, 0.97)	0.036	1.67% (1.50, 1.86)	0.83%
Favorable ICVHMs (*n* = 8108)	0.64	0.48 (0.30, 0.77)	0.002	0.80% (0.63, 1.01)	1.70%
Intermediate genetic risk					
ICVHMs per point		0.82 (0.74, 0.91)	0.001		
Unfavorable ICVHMs (*n* = 810)	2.77	1.00		2.54% (1.48, 4.33)	
Intermediate ICVHMs (*n* = 23,889)	1.75	0.71 (0.41, 1.21)	0.205	1.58% (1.40, 1.80)	0.96%
Favorable ICVHMs (*n* = 9481)	0.65	0.41 (0.22, 0.77)	0.005	0.51% (0.36, 0.72)	2.03%
Low genetic risk					
ICVHMs per point		0.87 (0.80, 0.94)	0.037		
Unfavorable ICVHMs (*n* = 794)	2.27	1.00		2.25% (1.44, 3.52)	
Intermediate ICVHMs (*n* = 22,534)	1.50	0.91 (0.59, 1.41)	0.674	1.48% (1.34, 1.64)	0.77%
Favorable ICVHMs (*n* = 8878)	0.79	0.71 (0.44, 1.15)	0.163	0.68% (0.54, 0.86)	1.57%
UKB					
High genetic risk					
ICVHMs per point		0.81 (0.76, 0.85)	<0.001		
Unfavorable ICVHMs (*n* = 4298)	2.77	1.00		2.74% (2.23, 3.35)	
Intermediate ICVHMs (*n* = 71,430)	1.19	0.47 (0.38, 0.58)	<0.001	1.13% (1.05, 1.22)	1.61%
Favorable ICVHMs (*n* = 22,979)	0.75	0.37 (0.28, 0.47)	<0.001	0.68% (0.58, 0.80)	2.06%
Intermediate genetic risk					
ICVHMs per point		0.78 (0.74, 0.82)	<0.001		
Unfavorable ICVHMs (*n* = 5166)	2.69	1.00		2.51% (2.09, 3.01)	
Intermediate ICVHMs (*n* = 84,540)	1.17	0.48 (0.39, 0.58)	<0.001	1.11% (1.04, 1.19)	1.40%
Favorable ICVHMs (*n* = 27,462)	0.62	0.32 (0.25, 0.41)	<0.001	0.57% (0.48, 0.67)	1.94%
Low genetic risk					
ICVHMs per point		0.80 (0.77, 0.85)	<0.001		
Unfavorable ICVHMs (*n* = 5030)	2.36	1.00		2.18% (1.79, 2.65)	
Intermediate ICVHMs (*n* = 86,207)	1.09	0.51 (0.41, 0.63)	<0.001	1.04% (0.97, 1.12)	1.14%
Favorable ICVHMs (*n* = 28,670)	0.59	0.34 (0.26, 0.44)	<0.001	0.57% (0.48, 0.69)	1.61%

^a^Cox proportional hazard regression in the CKB adjusted for sex, education, marital status, and family histories of heart attack or stroke at baseline and stratified jointly by study area and age at baseline in the 5-year interval. Cox proportional hazard regression in the UKB adjusted for sex, education, marital status, and socioeconomic status and first 20 principal components of ancestry and stratified jointly by age at baseline in the 5-year interval. HR, hazard ratio; *CI*, confidence interval

^b^*P* value for trend calculated treating the three scores as continuous variables

^c^Genetic risk categories were defined according to a weighted polygenic risk score as low (lowest tertile), intermediate (intermediate tertile), and high (highest tertile)

^d^*ICVHMs*, ideal cardiovascular health metrics. The categories defined according to ideal cardiovascular health metrics as favorable (6 to 8), intermediate (3 to 5), and unfavorable (0 to 2)

No significant additive interaction between PRS and ICVHMs was observed as shown in Supplementary Table 5, indicating that the risk of HF attributed to the combination of increased genetic risk and low ICVHMs was similar to the addition of the risk associated with each factor (*RERI* and *95% CI* were 0.05 [−0.22, 0.33] in CKB and 0.04 [−0.14, 0.22] in UKB). The attributable HF risk proportions of joint effect were 3.36% (−13.67, 20.39) in CKB and 2.38% (−8.65, 13.41) in UKB to their interaction.

## Discussion

In these 2 large community-based cohorts, lower genetic risk and ICVHMs were independently provided protective effects on the risk of incident HF. Irrespective of genetic risk, favorable ICVHMs was associated with a lower HF risk compared to unfavorable ICVHMs.

This study is the first to report the associations of combined cardiometabolic factors in different genetic risk groups for HF. Previous studies have tended to focus only on lifestyle factors associated with heart failure, ignoring the influence of genetic stratification. Most studies of lifestyle–gene interactions have targeted outcomes in other cardiovascular diseases. Specifically, the non-significant interactions we found are in line with two previous reports which used UKB data to investigate interactions between PRS and lifestyle score (not including metabolic factors) for other cardiovascular diseases (atrial fibrillation, stroke, and hypertension) [19, 24]. Moreover, a study which involved 14,051 participants of the EPIC-Norfolk study during a mean follow-up of 11.5 years computed the interaction terms between lipoprotein(a) genotype (as estimated by the rs10455872 variant) and cardiovascular health score (including metabolic factors) categories for cardiovascular diseases (coronary heart disease or stroke) risk prediction and found no significant interaction [23]. In the present study using 2 large prospective cohorts, lifestyle factors combined with metabolic factors were selected to construct ICVHMs, and more variants rather than a single variant were selected to construct genetic scores for HF, which contained more comprehensive genetic risk information. Lifestyle or cardiovascular health score and genetic risk appeared to be independently associated with HF or other cardiovascular diseases. In other words, there is a general benefit to cardiovascular outcomes if people maintain an ideal lifestyle and metabolism regardless of genetic risk, highlighting the importance of acquired health behaviors and the necessary interventional control of clinical metabolic indicators for cardiovascular health.

HF is a clinical syndrome of fluid congestion and exercise intolerance due to cardiac dysfunction [35]. As for the heritability of HF, a small proportion of HF cases are attributable to monogenic cardiomyopathies [36], while existing genome-wide association studies have yielded only limited insights, leaving the observed heritability of HF largely unexplained. Heterogeneity in the etiology and clinical presentation of HF may reduce statistical potency [20, 21]. For ideal cardiovascular health, it is mechanistically plausible that it has a protective effect on HF. The acute pro-thrombotic, adrenergic, and pro-inflammatory properties of smoking contribute to atherosclerosis, while quitting smoking reverses smoking-related endothelial dysfunction, thereby reducing the risk of cardiovascular diseases [37, 38]. Physical activities have benefits on endothelial function, autonomic function, nitric oxide bioavailability, and progenitor cell mobilization [39, 40]. Modest alcohol use improves endothelial function and increases plasma atrial natriuretic peptide [41]. Associations of obesity, blood pressure, glucose, and total cholesterol with incident HF may be mediated through coronary heart disease, type 2 diabetes, left ventricular hypertrophy, and sleep apnea, which are all major risk factors for HF and the postulated mechanisms include increases in atherogenic lipids, cardiac preload and afterload, and neurohormonal disruption [42, 43].

To the best of our knowledge, this is the first study to examine the associations of a comprehensive cardiovascular health metrics profile in different genetic risk levels in the prevention of HF. This study was conducted in two large, independent prospective cohorts of different races. The large sample size and prospective design of the CKB and the UKB study provided strong and broadly applicable evidence. In this study, PRS for HF contained multiple genes based on GWAS. A more comprehensive ICVHMs was constructed with additional consideration for the effects of alcohol drinking and central obesity. Interaction between ICVHMs and genetic risk on the development of HF was tested both on the multiplicative and additive scale to get more convincing evidence. Although no significant gene–environment interaction was observed, our results underscore the importance of early interventions targeting combined lifestyle behaviors and cardiometabolic risk factors to prevent HF across Asian and European populations, regardless of genetic risk. In addition, we carefully controlled for potential confounders and excluded participants at baseline with major chronic diseases at baseline that might cause lifestyle changes to minimize the inverse causal biases.

This study has several limitations. First, causalities between the cardiovascular risk factors and HF cannot be inferred from the observational study design, but can be concluded from subsequent Mendelian randomization studies. Second, although we have adjusted for confounders and excluded people with significant cardiovascular events at baseline, unmeasured confounders and reverse causation remain. Third, the lifestyle factors were self-reported once at baseline and might not necessarily reflect the long-term patterns. Possible measurement and classification errors are likely biased toward the null and would underestimate the risk associated with poor health behaviors and factors. Fourth, different ways of measurement and definition in the two cohorts made the results not much comparable between the two populations. As a result of the difference in guideline recommendations, the classification boundaries for alcohol consumption, BMI, and waist circumference were different. In CKB, the lack of fasting glucose data was replaced by random blood glucose; ideal lipid levels were defined as non-use of lipid-lowering drugs because lipid was not measured at baseline; and food intake was measured only by the frequency of intake in the questionnaire. Each of these definitions applies to a single population, so for interpretation of the results of this study, we should focus on the results in their respective populations. Fifth, the SNPs used to construct PRS were not found in GWAS of the Asian population and may also have pleiotropic effects on cardiovascular risk factors. In this study, we used genome-wide significant SNPs for heart failure in the European population to construct PRS in the Asian population. Only two SNPs were significantly associated with heart failure in CKB, limiting the power to detect effects. And the GWAS population we used to build PRS included the UKB population, which might cause results biased due to winner’s curse. Therefore, we expect that more HF-related genetic variants in Asians and other Europeans will be identified in future larger GWAS; thus, variation explained by genetics and genetic risk estimates will be improved.

## Conclusions

In conclusion, our data show that genetic risk and cardiovascular health metrics were independently associated with the risk of incident HF in both the Chinese and European populations, which highlights the importance of adhering to favorable cardiovascular health in preventing the development of HF across all gradients of genetic risk.

## Supplementary Information

**Additional file 1: Figure S1-2, & Table S1-6, & Members of the China Kadoorie Biobank collaborative group. Fig S1** - Distribution of the Polygenic Risk Score (PRS) in the CKB. **Fig S2** - Distribution of the Polygenic Risk Score (PRS) and the Incidence of Heart Failure according to PRS in the UKB. **Table S1** - Significant SNPs of heart failure from genome-wide association studies. **Table S2** - Risk of Incident Heart Failure According to Individual SNPs in the CKB. **Table S3** - Healthy Lifestyle Factor Definitions in CKB and UKB. **Table S4** - Risk of Incident Heart Failure in the CKB According to Genetic Risk based on all 15 SNPs from the Previous GWAS. **Table S5** - P values for interaction based on the multiplicative and additive effects model in the CKB and UKB. **Table S6** - Risk of Incident Heart Failure According to Genetic (based on all 15 SNPs from the Previous GWAS) and Cardiovascular Health Metrics Risk in CKB. Members of the China Kadoorie Biobank collaborative group.

## Data Availability

Details of how to access China Kadoorie Biobank data and details of the data release schedule are available from www.ckbiobank.org/site/Data+Access. UKB data are available in a public, open access repository. This research has been conducted using the UK Biobank Resource under Application Number 44430. The UK Biobank data are available on application to the UK Biobank (www.ukbiobank.ac.uk/).

## Abbreviations

AHA: American Heart Association
AP: Attributable proportion due to interaction
*BMI*: Body mass index
*CI*: Confidence interval
CKB: China Kadoorie Biobank
GWAS: Genome-wide association studies
HF: Heart failure
*HR*: Hazard ratio
ICD: International Classification of Diseases
ICVHMs: Idea cardiovascular health metrics
PRS: Polygenic risk score
*RERI*: Relative excess risk due to interaction
SD: Standard deviation
SNP: Single nucleotide polymorphism
UKB: UK Biobank
WC: Waist circumference
