# Supplementary material for: Modification effect of ideal cardiovascular health metrics on genetic association with incident heart failure in the China Kadoorie Biobank and the UK Biobank
Source: BMC Med. 2021 Oct 22;19:259. doi: 10.1186/s12916-021-02122-1 (PMC8532287; doi:10.1186/s12916-021-02122-1)
Supplement: Supplementary file 1 — Additional file 1: Figure S1-2, & Table S1-6, & Members of the China Kadoorie Biobank collaborative group. Fig S1 - Distribution of the Polygenic Risk Score (PRS) in the CKB. Fig S2 - Distribution of the Polygenic Risk Score (PRS) and the Incidence of Heart Failure according to PRS in the UKB. Table S1 - Significant SNPs of heart failure from genome-wide association studies. Table S2 - Risk of Incident Heart Failure According to Individual SNPs in the CKB. Table S3 - Healthy Lifestyle Factor Definitions in CKB and UKB. Table S4 - Risk of Incident Heart Failure in the CKB According to Genetic Risk based on all 15 SNPs from the Previous GWAS. Table S5 - P values for interaction based on the multiplicative and additive effects model in the CKB and UKB. Table S6 - Risk of Incident Heart Failure According to Genetic (based on all 15 SNPs from the Previous GWAS) and Cardiovascular Health Metrics Risk in CKB. Members of the China Kadoorie Biobank collaborative group. [file 12916_2021_2122_MOESM1_ESM.docx]

**Supplementary Material**

Supplementary Figures (2)

Fig S1 - Distribution of the Polygenic Risk Score (PRS) in the CKB.

Fig S2 - Distribution of the Polygenic Risk Score (PRS) and the Incidence of Heart Failure according to PRS in the UKB.

Supplementary Tables (6)

Table S1 - Significant SNPs of heart failure from genome-wide association studies

Table S2 - Risk of Incident Heart Failure According to Individual SNPs in the CKB.

Table S3 - Healthy Lifestyle Factor Definitions in CKB and UKB.

Table S4 - Risk of Incident Heart Failure in the CKB According to Genetic Risk based on all 15 SNPs from the Previous GWAS.

Table S5 - P values for interaction based on the multiplicative and additive effects model in the CKB and UKB.

Table S6 - Risk of Incident Heart Failure According to Genetic (based on all 15 SNPs from the Previous GWAS) and Cardiovascular Health Metrics Risk in CKB.

Members of the China Kadoorie Biobank collaborative group

Fig S1. Distribution of the Polygenic Risk Score (PRS) in the CKB.


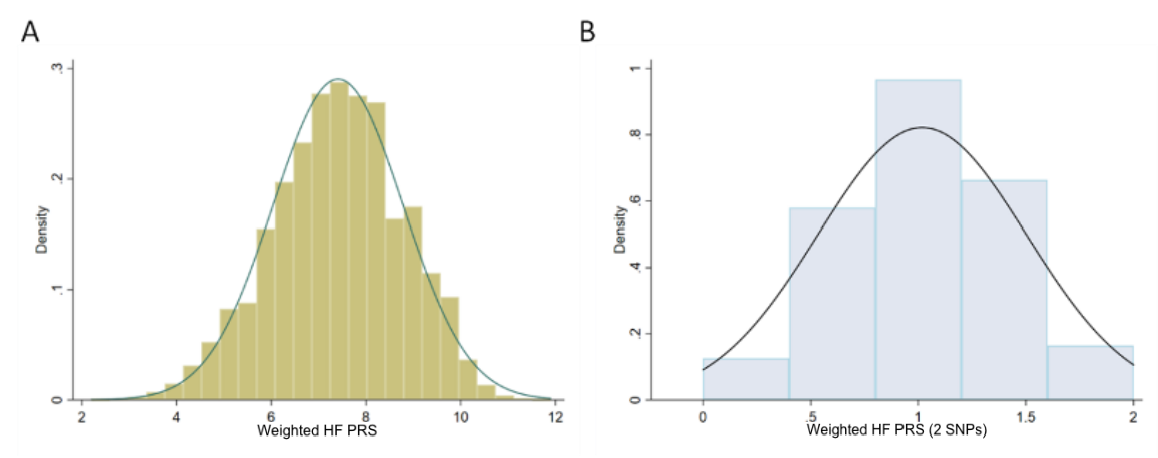


A, Distribution of PRS using 15 SNPs from the previous GWAS of heart failure in the CKB testing dataset (n=92 602). The x axis represents PRS.

B, Distribution of PRS using 2 SNPs which are significant in the CKB from the previous GWAS of heart failure in the CKB testing dataset (n=92 602). The x axis represents PRS.

Fig S2. Distribution of the Polygenic Risk Score (PRS) and the Incidence of Heart Failure according to PRS in the UKB.


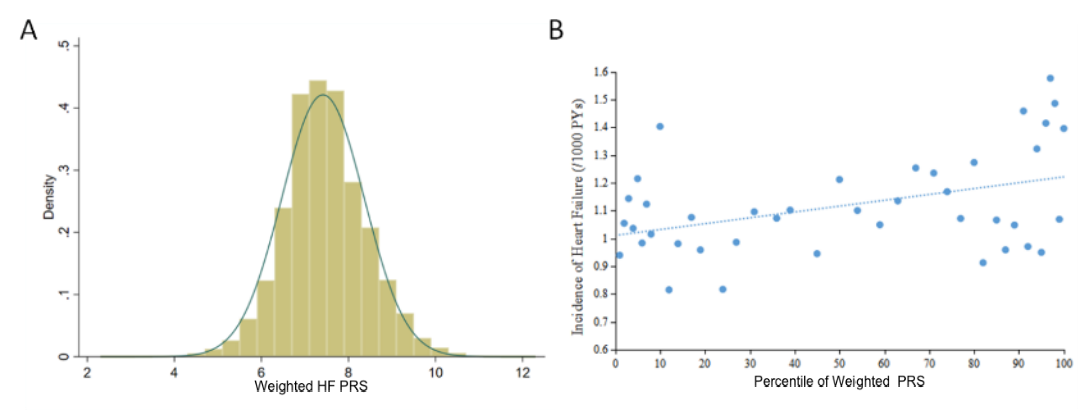


A, Distribution of PRS of heart failure in the UK Biobank testing dataset (n= 335 782). The x axis represents PRS.

B, Incidence of heart failure according to 100 groups of the testing dataset binned according to the percentile of the GPS. PYs, person-years.

Table S1. Significant SNPs of heart failure from genome-wide association studies

| **rsID** | **Position (hg19)** | **Nearest gene (s)^a^** | **Risk/ref allele** | **RAF (%)** | **OR (95% CI)** | **P value** |
| --- | --- | --- | --- | --- | --- | --- |
| rs660240 | 109817838 | CELSR2 | C/T | 0.79 | 1.06 (1.04, 1.08) | 3.25E-10 |
| rs73839819 | 72579834 | RYBP | A/G | 0.02 | 1.33 (1.19, 1.48) | 2.65E-07 |
| rs17042102 | 111668626 | PITX2, FAM241A | A/G | 0.12 | 1.12 (1.09, 1.14) | 5.71E-20 |
| rs1906609 | 111666451 | PITX2 | T/G | 0.16 | 1.15 (1.10, 1.21) | 9.08E-10 |
| rs11745324 | 137012171 | KLHL3 | G/A | 0.77 | 1.05 (1.03, 1.07) | 2.35E-08 |
| rs4135240 | 36647680 | CDKN1A | T/C | 0.66 | 1.05 (1.03, 1.07) | 6.84E-09 |
| rs55730499 | 161005610 | LPA | T/C | 0.07 | 1.11 (1.08, 1.14) | 1.83E-11 |
| rs140570886 | 161013013 | LPA | C/T | 0.02 | 1.24 (1.16, 1.30) | 7.69E-11 |
| rs1556516 | 22100176 | 9p21/CDKN2B-AS1 | C/G | 0.48 | 1.06 (1.05, 1.08) | 1.57E-15 |
| rs600038 | 136151806 | ABO, SURF1 | C/T | 0.21 | 1.06 (1.04, 1.08) | 3.68E-09 |
| rs4746140 | 75417249 | SYNPO2L, AGAP5 | G/C | 0.85 | 1.07 (1.05, 1.09) | 1.10E-09 |
| rs17617337 | 121426884 | BAG3 | C/T | 0.78 | 1.06 (1.04, 1.08) | 3.65E-09 |
| rs4766578 | 111904371 | ATXN2 | T/A | 0.47 | 1.04 (1.03, 1.06) | 4.90E-08 |
| rs56094641 | 53806453 | FTO | G/A | 0.42 | 1.05 (1.03, 1.06) | 1.21E-08 |
| rs12627426 | 30519457 | MAP3K7CL | T/A | 0.16 | 1.13 (1.08, 1.18) | 2.63E-07 |

Chr, chromosome; ref, reference; RAF, risk allele frequency; OR, odds ratio; CI, confidence intervals

a Nearest gene with a functional protein or RNA (e.g., anti-sense RNA) product that either overlaps with the sentinel variant, or for intergenic variants, the nearest genes up- and downstream, respectively (separated by comma)

Table S2. Risk of Incident Heart Failure According to Individual SNPs in the CKB.

| rsID | Risk/ref allele | Beta | 95% CI | P value |
| --- | --- | --- | --- | --- |
| rs660240 | C/T | 0.05 | (-0.11, 0.20) | 0.571 |
| rs73839819 | A/G | 0.01 | (-0.06, 0.09) | 0.756 |
| rs1906609 | A/G | 0.07 | (-0.02, 0.15) | 0.122 |
| **rs17042102** | T/G | 0.10 | (0.02, 0.17) | **0.010** |
| rs11745324 | G/A | -0.01 | (-0.17, 0.16) | 0.917 |
| rs4135240 | T/C | 0.08 | (0.00, 0.17) | 0.064 |
| rs55730499 | T/C | 0.03 | (-1.77, 1.71) | 0.973 |
| rs140570886 | C/T | -0.19 | (-0.44, 0.06) | 0.136 |
| rs1556516 | C/G | 0.02 | (-0.06, 0.10) | 0.609 |
| rs600038 | C/T | 0.05 | (-0.03, 0.14) | 0.218 |
| **rs4746140** | G/C | 0.08 | (0.01, 0.16) | **0.036** |
| rs17617337 | C/T | 0.13 | (-0.54, 0.79) | 0.713 |
| rs4766578 | T/A | 0.08 | (-0.50, 0.67) | 0.781 |
| rs56094641 | G/A | 0.07 | (-0.04, 0.18) | 0.198 |
| rs12627426 | T/A | -0.05 | (-0.14, 0.04) | 0.261 |

Cox proportional hazards regression adjusted for sex, education, marital status and family histories of heart attack or stroke at baseline and stratified jointly by study area and age at baseline in 5-year interval.

Table S3. Healthy Lifestyle Factor Definitions in CKB and UKB.

| **Lifestyle** | **Healthy group in CKB** | **Healthy group in UKB** |
| --- | --- | --- |
| Smoking | Never smoker  Occasional smoker  Ex-smoker (stop smoking not due to illness) | No current smoking |
|  |  |  |
| Alcohol consumption | Never-regular drinker  Ex-regular drinker  Weekly drinker  Moderate daily drinker (≤25 g of pure alcohol in men and ≤15 g in women per day) | Moderate daily drinker (>0 and ≤28g of pure alcohol in men and >0 and ≤14g in women per day) |
|  |  |  |
| Diet | Those who ate vegetables and fruit daily and red meat on one to six days a week | At least 4 of the following 5 foods:  Fruits: ≥ 3 servings/day Vegetables: ≥ 3 servings/day Fish: ≥2 servings/week Processed meats: ≤ 1 serving/week Unprocessed red meats: ≤ 1.5 servings/week |
|  |  |  |
| Physical activity | Those whose total physical activity levels were above the sex-specific median level | ≥150 minutes moderate activity per week or ≥ 75 minutes vigorous activity per week or equivalent combination or moderate physical activity at least 5 days a week or vigorous activity once a week |
|  |  |  |
| Obesity | 18.5 kg/m^2^<BMI < 24.0 kg/m^2^  WC<90cm in men and <85 cm in women | 18.5kg/m^2^<BMI<24.0kg/m^2^, WC<102cm in men and <88 cm in women |

Table S4. Risk of Incident Heart Failure in the CKB According to Genetic Risk based on all 15 SNPs from the Previous GWAS.

| Genetic Risk (15 SNPs) | Cases/1 000 PYs | HR (95% CI) | P trend |
| --- | --- | --- | --- |
| Low (n=31 753) | 1.31 | 1.00 | 0.067 |
| Intermediate (n=33 650) | 1.45 | 1.12 (0.99, 1.27) |  |
| High (n=29 160) | 1.48 | 1.14 (1.00, 1.30) |  |

Table S5. P values for interaction based on the multiplicative and additive effects model in the CKB and UKB.

|  | CKB |  | UKB |
| --- | --- | --- | --- |
| Multiplicative effects model (P_int_) | 0.616 |  | 0.726 |
| Additive effects model |  |  |  |
| RERI | 0.05 (-0.22, 0.33) |  | 0.04 (-0.14, 0.22) |
| AP, % | 3.36 (-13.67, 20.39) |  | 2.38 (-8.65, 13.41) |

Table S6. Risk of Incident Heart Failure According to Genetic (based on all 15 SNPs from the Previous GWAS) and Cardiovascular Health Metrics Risk in CKB.

|  | Cases/1 000 PYs | HR (95% CI) |
| --- | --- | --- |
| Low Genetic Risk (15 SNPs) |  |  |
| Favorable ICVHMs (n=11 520) | 0.79 | 1.00 |
| Intermediate ICVHMs (n=92 403) | 1.50 | 1.11 (0.86, 1.42) |
| Unfavorable ICVHMs (n=15 870) | 2.27 | 1.33 (0.79, 2.24) |
| Intermediate Genetic Risk |  |  |
| Favorable ICVHMs (n=9 481) | 0.65 | 0.84 (0.61, 1.16) |
| Intermediate ICVHMs (n=898 957) | 1.75 | 1.30 (1.02, 1.66) |
| Unfavorable ICVHMs (n=15 982) | 2.77 | 1.77 (1.10, 2.87) |
|  |  |  |
| High Genetic Risk |  |  |
| Favorable ICVHMs (n=9 213) | 0.64 | 0.83 (0.59, 1.17) |
| Intermediate ICVHMs (n=75 900) | 1.77 | 1.33 (1.04, 1.70) |
| Unfavorable ICVHMs (n=13 512) | 3.04 | 1.81 (1.12, 2.93) |

**Members of the China Kadoorie Biobank collaborative group:**

**International Steering Committee:** Junshi Chen, Zhengming Chen (PI), Robert Clarke, Rory Collins, Yu Guo, Liming Li (PI), Jun Lv, Richard Peto, Robin Walters. **International Co-ordinating Centre, Oxford:** Daniel Avery, Ruth Boxall, Derrick Bennett, Yumei Chang, Yiping Chen, Zhengming Chen, Robert Clarke, Huaidong Du, Simon Gilbert, Alex Hacker, Mike Hill, Michael Holmes, Andri Iona, Christiana Kartsonaki, Rene Kerosi, Ling Kong, Om Kurmi, Garry Lancaster, Sarah Lewington, Kuang Lin, John McDonnell, Iona Millwood, Qunhua Nie, Jayakrishnan Radhakrishnan, Paul Ryder, Sam Sansome, Dan Schmidt, Paul Sherliker, Rajani Sohoni, Becky Stevens, Iain Turnbull, Robin Walters, Jenny Wang, Lin Wang, Neil Wright, Ling Yang, Xiaoming Yang. **National Co-ordinating Centre, Beijing:** Yu Guo, Xiao Han, Can Hou, Jun Lv, Pei Pei, Chao Liu, Canqing Yu. **10 Regional Co-ordinating Centres: Qingdao CDC:** Zengchang Pang, Ruqin Gao, Shanpeng Li, Shaojie Wang, Yongmei Liu, Ranran Du, Yajing Zang, Liang Cheng, Xiaocao Tian, Hua Zhang, Yaoming Zhai, Feng Ning, Xiaohui Sun, Feifei Li. **Licang CDC:** Silu Lv, Junzheng Wang, Wei Hou. **Heilongjiang Provincial CDC:** Mingyuan Zeng, Ge Jiang, Xue Zhou. **Nangang CDC:** Liqiu Yang, Hui He, Bo Yu, Yanjie Li, Qinai Xu,Quan Kang, Ziyan Guo. **Hainan Provincial CDC:** Dan Wang, Ximin Hu, Jinyan Chen, Yan Fu, Zhenwang Fu, Xiaohuan Wang. **Meilan CDC:** Min Weng, Zhendong Guo, Shukuan Wu,Yilei Li, Huimei Li, Zhifang Fu. **Jiangsu Provincial CDC:** Ming Wu, Yonglin Zhou, Jinyi Zhou, Ran Tao, Jie Yang, Jian Su. **Suzhou CDC:** Fang liu, Jun Zhang, Yihe Hu, Yan Lu, Liangcai Ma, Aiyu Tang, Shuo Zhang, Jianrong Jin, Jingchao Liu. **Guangxi Provincial CDC:** Zhenzhu Tang, Naying Chen, Ying Huang. **Liuzhou CDC:** Mingqiang Li, Jinhuai Meng, Rong Pan, Qilian Jiang, Jian Lan,Yun Liu, Liuping Wei, Liyuan Zhou, Ningyu Chen Ping Wang, Fanwen Meng, Yulu Qin,, Sisi Wang. **Sichuan Provincial CDC:** Xianping Wu, Ningmei Zhang, Xiaofang Chen,Weiwei Zhou. **Pengzhou CDC:** Guojin Luo, Jianguo Li, Xiaofang Chen, Xunfu Zhong, Jiaqiu Liu, Qiang Sun. **Gansu Provincial CDC:** Pengfei Ge, Xiaolan Ren, Caixia Dong. **Maiji CDC:** Hui Zhang, Enke Mao, Xiaoping Wang, Tao Wang, Xi zhang. **Henan Provincial CDC:** Ding Zhang, Gang Zhou, Shixian Feng, Liang Chang, Lei Fan. **Huixian CDC:** Yulian Gao, Tianyou He, Huarong Sun, Pan He, Chen Hu, Xukui Zhang, Huifang Wu, Pan He. **Zhejiang Provincial CDC:** Min Yu, Ruying Hu, Hao Wang. **Tongxiang CDC:** Yijian Qian, Chunmei Wang, Kaixu Xie, Lingli Chen, Yidan Zhang, Dongxia Pan, Qijun Gu. **Hunan Provincial CDC:** Yuelong Huang, Biyun Chen, Li Yin, Huilin Liu, Zhongxi Fu, Qiaohua Xu. **Liuyang CDC:** Xin Xu, Hao Zhang, Huajun Long, Xianzhi Li, Libo Zhang, Zhe Qiu.
